# Automated Segmentation Tool for Brain Infusions

**DOI:** 10.1371/journal.pone.0064452

**Published:** 2013-06-05

**Authors:** Kathryn Hammond Rosenbluth, Francisco Gimenez, Adrian P. Kells, Ernesto A. Salegio, Gabriele M. Mittermeyer, Kevin Modera, Anmol Kohal, Krystof S. Bankiewicz

**Affiliations:** Department of Neurosurgery, University of California San Francisco (UCSF), San Francisco, California, United States of America; Boston Children's Hospital, United States of America

## Abstract

This study presents a computational tool for auto-segmenting the distribution of brain infusions observed by magnetic resonance imaging. Clinical usage of direct infusion is increasing as physicians recognize the need to attain high drug concentrations in the target structure with minimal off-target exposure. By co-infusing a Gadolinium-based contrast agent and visualizing the distribution using real-time using magnetic resonance imaging, physicians can make informed decisions about when to stop or adjust the infusion. However, manual segmentation of the images is tedious and affected by subjective preferences for window levels, image interpolation and personal biases about where to delineate the edge of the sloped shoulder of the infusion. This study presents a computational technique that uses a Gaussian Mixture Model to efficiently classify pixels as belonging to either the high-intensity infusate or low-intensity background. The algorithm was implemented as a distributable plug-in for the widely used imaging platform OsiriX®. Four independent operators segmented fourteen anonymized datasets to validate the tool’s performance. The datasets were intra-operative magnetic resonance images of infusions into the thalamus or putamen of non-human primates. The tool effectively reproduced the manual segmentation volumes, while significantly reducing intra-operator variability by 67±18%. The tool will be used to increase efficiency and reduce variability in upcoming clinical trials in neuro-oncology and gene therapy.

## Introduction

Neuro-imaging can be used to non-invasively assess the performance of drug infusions in the brain. Accurate characterization of the infusate distribution in the brain parenchyma is critical to evaluating the success of an infusion on a patient-by-patient basis. Such characterization provides crucial information to improve future infusion protocols and understand the success or failure of clinical trials. In fact, visualization is so useful that clinical trials are moving beyond post-operative to intra-operative magnetic resonance imaging (MRI) to monitor the drug distribution in real-time. A suite of technologies has been developed to support trials using direct pressure-driven brain infusions, often called Convection Enhanced Delivery (CED) [1]. These technologies have included image-guided stereotaxy [2], improved cannulae [2], [3] and surgical planning software [4], [5]. CED achieves high concentrations of drug with minimal off-target exposure since it bypasses the blood-brain barrier and displaces the interstitial fluid, thus making it possible to deliver large macromolecules with pinpoint accuracy. These technologies should help overcome the poor drug distributions observed in several recent high-profile clinical trials involving direct delivery of neurotrophic factors [6], [7] or gene therapy vectors for the treatment of Parkinson’s disease [8], [9] and of immunotoxins [10], [11] or chemotherapies [12] for treating brain tumors.

By co-infusing Gadolinium as a MRI-visible surrogate tracer, clinicians can evaluate the drug distribution in real-time and make informed decisions regarding when to terminate the infusion or to adjust the position of the infusion cannula to optimize target coverage. Gadolinium produces high intensity signals on T1-weighted images. However, manual segmentation of these images is tedious and affected by window settings, image interpolation, variation in MRI contrast, MRI noise, and subjective interpretations of the sloped edge of the infusion. Reported ratios between the distribution volume (*Vd*) measured in the MRI images and the infusion volume reported by the pump (*Vi*) have previously ranged from as low as 1.5 [13] to greater than 5 [14], [15], [16]. Unfortunately, it is difficult to ascertain whether these differences result from true variability between infusions (for example, due to the leakage of infusate) or whether the differences result from operator bias in the image segmentation. Hence, a computational-based approach to delineate infusion volumes would be preferable to increase efficiency and reduce variability in segmentation in upcoming clinical trials.

The goal of this study was to develop an autosegmentation technique that identified similar volumes to expert operators, reduced the inter-operator variability, ran quickly and was robust to variables such as noise, windowing, or resolution. The technique was based on a Gaussian Mixture Model pixel classification [17] that modeled the pixel distribution as containing two distinct functions: a high intensity function of infused pixels and a low intensity function of non-infused pixels. The technique was built as a plug-in for OsiriX®, a widely distributed imaging platform available from the NIH. The technique was retrospectively validated using T1-weighted images of Gadolinium infusions into the brain of non-human primates (NHP).

## Methods

### Semi-Automated Infusion Segmentation by Gaussian Mixture Model

**Figure 1:**
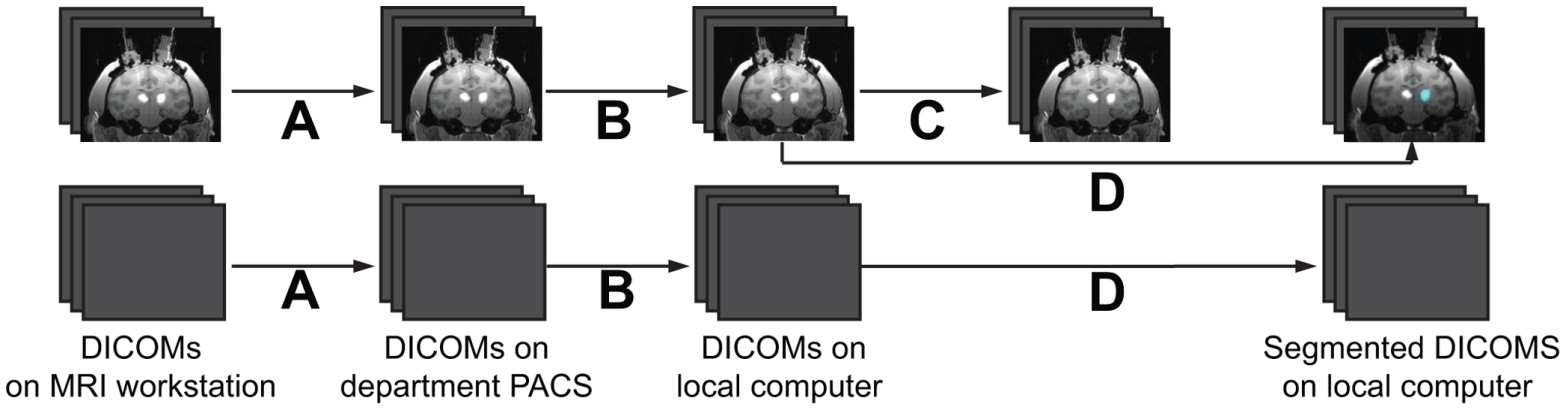
System architecture and implementation. MRI DICOM volumes were acquired every 5 minutes. (A) New DICOM volumes were automatically pushed to the department PACS for archiving by the GE DICOM Sender. (B) The operator manually pulled and anonymized DICOM images on a local laptop using the Osirix PACS Query/Retrieve tool. (C) The operator recorded the location of a three-dimensional box on the infusion target region in one image volume. (D) The user used the segmentation plug-in to automatically segment the infusion in volume. The segmentation was many times faster (<1 second) than the MRI data acquisition (∼5 minutes) and data transfer (∼1 minute).



The segmentation was written in Objective-C (Cocoa) and implemented as an OsiriX® plugin for easy usability (OsiriX® Medical Image Software, v3.9.1; Geneva, Switzerland). The system architecture and implementation is shown in Figure 1. DICOM (Digitial Imaging and Communications in Medicine) formatted MRI images were pushed from the MRI workstation, to the department PACS (Pictures Archiving and Communication System), to a local computer running Osirix®. Segmentation using the plug-in was done on the local computer (MacBook Pro, Mac OS X Version 10.7.5, Processor: 2.8 GHz Intel Core i7, Memory: 4GB 1333 MHz DDR3, Graphics: Intel HD Graphics 3000 384 MB). The pixels in each three-dimensional image volume were normalized to the range of values in the scan prior to classification:
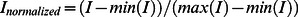
(1)and each pixel was classified as belonging to either the class of infused pixels (foreground) or non-infused pixels (background). The classes were modeled as having additive Gaussian noise, producing Normal distribution functions (*N)* parameterized by the mean background value (*μ_0_*), mean foreground value (*μ_1_*), standard deviation of background noise (*σ_0_*), and standard deviation of foreground noise (*σ_1_*):

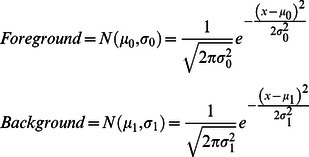
(2)Assuming the likelihood that each pixel drawn from the foreground was *α*, the joint probability density function of pixel intensities was modeled as (2):
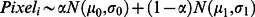
(3)


This combination of Gaussian distributions is called a Gaussian Mixture Model (GMM), where α is referred to as the mixing parameter.

The Expectation Maximization (EM) algorithm [18], [19], [20] was used to determine the five parameters of the model: *α, μ_0_, σ_0_, μ_1_,* and *σ_1_*. EM is an efficient, iterative algorithm to estimate model parameters from data with missing values. In this case, the missing data was the class membership of each pixel. EM finds the best solution by alternating between estimating the values of the missing data using their expected valu and computing the maximum likelihood values of the model parameters with those estimated values. The algorithm iterates until the change in the fit drops below a predefined tolerance level. Model fit was calculated using the negative log-likelihood:
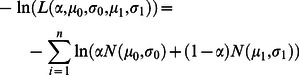
(4)


Starting values for the parameters were *μ_0_* = .2, *σ_0_* = .2, *μ_1_* = .8, *σ_1_* = .2 and *α = *0.5. The algorithm was iterated until the effect of modifying parameters dropped below a threshold 0.1%. Each pixel was then classified by calculating whether there was a higher probability that it belonged to the foreground or background class.

Three-dimensional morphological closing and opening with spheroidal elements 5 by 5 by 3 pixels wide were used to correct scattered pixels causing holes in the infusion volume or isolated pixels in the background. Infusions produce large contiguous volumes, so geometrically disconnected pixels were assumed to have been erroneously classified due to noise or image artifacts such as zipper artifacts from radiofrequency interference or flow artifacts from the carotid arteries.

### Data Acquisition

CED infusions of saline doped with Gadoteridol (1.0 mM Gd-DTPA, Prohance: Bracco Diagnostics, Princeton, NJ) were conducted in fourteen NHP targeting either the putamen (N = 10, Vi = 50.4±6.5 µL) or thalamus (N = 4, Vi = 240.2±70.7 µL). The infusate also contained 2.3e12 vg/ml of the gene therapy vector AAV2-GDNF, which was being evaluated pre-clinically as a treatment for Parkinson’s disease [21], [22]. The experiments were performed according to National Institutes of Health guidelines and to protocols approved by the Institutional Animal Care and Use Committee at University of California San Francisco (San Francisco, CA). Animals were housed in a temperature and humidity controlled environment with a 12 hour light/dark cycle. Primate chow and water were available at all times. Enrichment was provided by providing chew and play toys in the cages and offering a variety of fruit and vegetables. Animals were monitored at least twice daily for the duration of the study, in addition to periodic behavioral assessments. The use of NHP was deemed necessary because NHP provided the closest comparable to humans for the surgery, imaging and drug delivery. To minimize suffering, the animals were sedated with ketamine (Ketaset, 7 mg/kg, intramuscular) and xylazine (Rompum, 3 mg/kg, intramuscular) during the surgery and with 1–3% inhaled isoflurane during the infusion.

The infusion protocol and image-guided infusion platform has been previously described [2]. Briefly, the infusion cannula were inserted into the target under image guidance by passing the cannula through a small burr-hole in the skull. The infusion was ramped up by 0.5 µL/min every 5 min up to a rate of 5 µL/min. Serial T1-weighted fast low angle shot (FLASH) images were acquired every 5 min to visualize the infusion (4.49 ms TE, 17 ms TR, 40° flip angle, 2 repetitions, 0.7 mm in-plane resolution, 180 mm field of view, 1 mm slices).

### Semi-Blinded Comparison of Segmented Volumes

Four operators independently completed manual segmentation and automated segmentation of the 14 anonymized datasets. The operators were experienced with manually segmenting Gadoteridol infusion on T1 images in OsiriX® and were given instructions and training in operating the autosegmentation plug-in tool. The operators were first asked to perform manual segmentation on all infusions. They were then asked to draw a three-dimensional bounding box around the infusion volumes to run the GMM classification. The corners of the bounding box were used to restrict the GMM classification to pixels inside the box. The bounding box was used to speed up processing, to differentiate between multiple infusions on the same scan and to eliminate bright structures in the background, such as fat. Once the bounded region was selected, the GMM classification was run and the resulting segmentation results were recorded.

### Statistics

Inter-operator variability was measured using the coefficient of variance (CoV). CoV was calculated as the ratio of the standard deviation between operators to the mean of the operators. Statistical significance was evaluated using Pearson’s correlation coefficient.

## Results and Discussion

**Figure 2:**
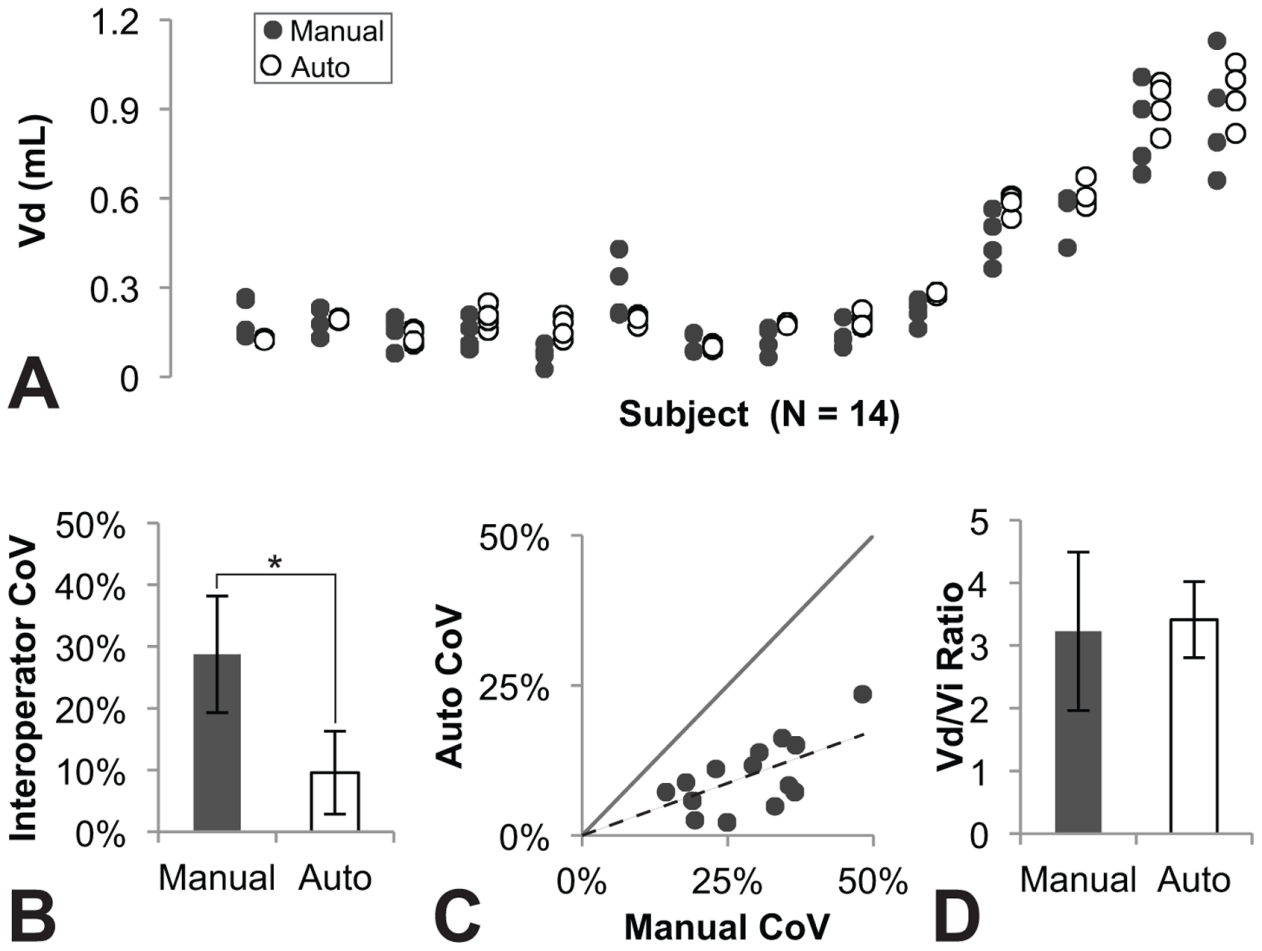
Comparison of manually segmented infusion volumes to autosegmented infusion volumes. (A) Autosegmentation (open dots) by the four operators produced consistent volumes to manual segmentation (black dots) in all fourteen test subjects. (B) Autosegmentation significantly reduced the interoperator variability (Mean ± StDev). (C) Autosegmentation variability for all studies was smaller than manual segmentation variability, so all values fell below the unity line. (D) Autosegmentation produced consistent Vd/Vi ratios to manual segmentation with lower standard deviation (Mean ± StDev).

The semi-automated tool provided more consistent segmentations than manual segmentations and had similar volumes to the manual segmentations (Figure 2A). Use of the autosegmentation tool significantly reduced inter-operator variability between the four operators from 29±9% to 10±6% (p<1.8E-7)(Figure 2B). Variability was improved in all tests cases (Figure 2C), with an average improvement of 67±18%. The average autosegmentation volumes for each study were closely correlated to the average manually segmented volumes (R^2^ = 0.957), making the *Vd/Vi* ratios more consistent between operators and across individual animals (Figure 2D).

**Figure 3:**
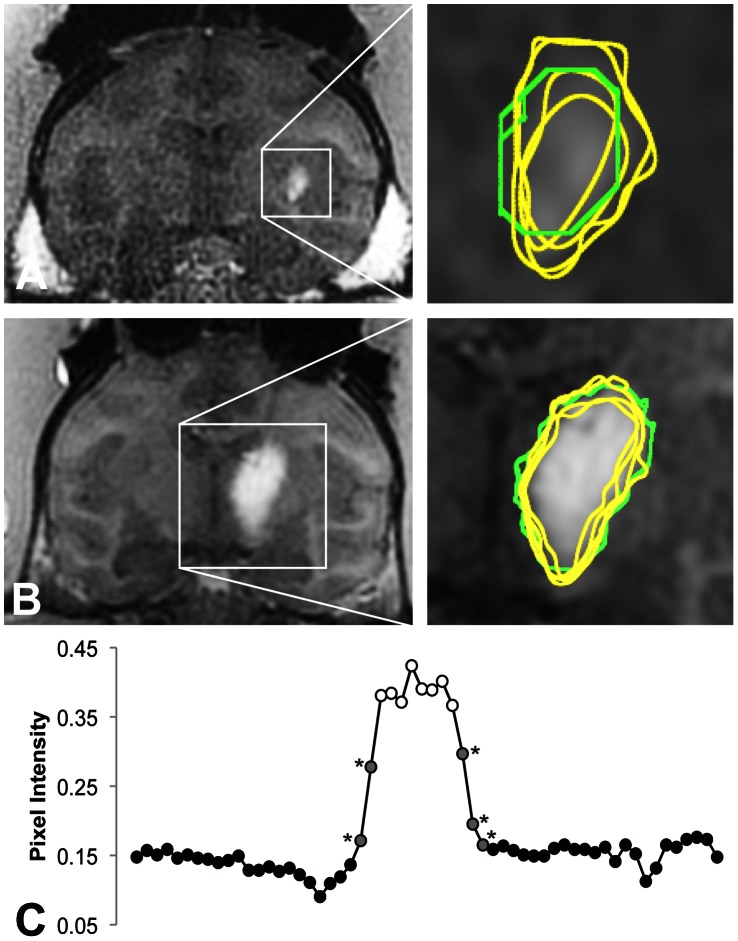
Representative manual (yellow) and automated (green) segmentations of the T1-weighted FLASH images. The automated segmentations were more consistent in both a (A) a 50 µL infusion in the putamen (manual: 177/226/131/232 µL; automated: 156/154/161/154 µL) and (B) a 160 µL infusion in the thalamus (manual: 425/505/364/564 µl; automated: 402/454/522/497 µL). (C) A cross-section of the thalamus infusion along the dotted line demonstrated that the inconsistently classified pixels (gray, starred) were on the shoulder of the infusion.

The examples in Figure 3 demonstrated the improved consistency of autosegmentation. The inter-operator CoV of manual segmentations was 25% in the putamen example (Figure 3A) and 19% in the thalamus example (Figure 3B). The autosegmentation tool reduced the inter-operator CoVs to 2% and 6%, respectively. The pixel intensity plot shown in (Figure 3C) demonstrated the intensity slope at the edge of the hyperintense infusate region. Operator bias in deciding where to place the edge of the segmentation on this slope led to the high variability in manual segmentations.

**Figure 4:**
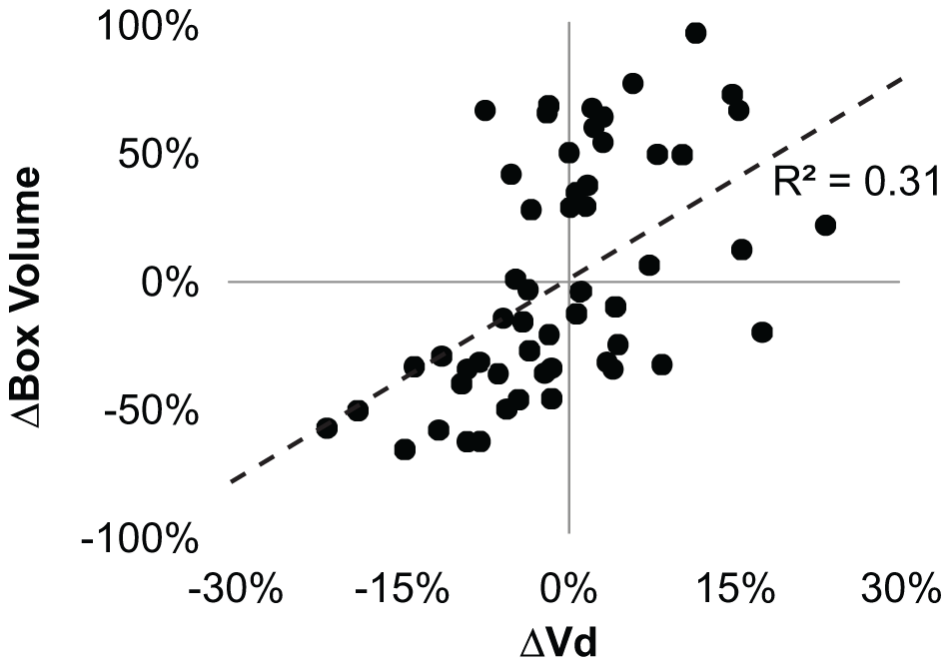
Influence of the operator-defined box on the autosegmentation volume. The difference between the individual and average box volumes was weakly correlated to the difference between the individual and average autosegmentation volume. However, there was considerably greater spread in the box volumes (165%) than autosegmentation volumes (55%).

The tool reduced but did not entirely eliminate the inter-operator CoV (Figure 2B–C). The residual variability in the autosegmentation volumes was weakly correlated with the volume of the operator-defined box (R^2^ = 0.31; Figure 4), suggesting that either the number or intensity values of the background pixels influenced the pixel classification. The mixture model was imperfect in its assumption that the background and foreground each had one homogeneous value, when in fact, the background contained brain structures that varied in their signal characteristics and the foreground contained a range of values influenced by the local concentration of Gadoteridol (Figure 3C). The correlation was not strong enough to lead to significant statistical differences in autosegmentation volumes between operators, despite significant differences in box sizes. For example, the first operator drew boxes twice as large as the second operator (averaging 4227 and 2229 mm^3^, respectively; p<1.3E-4), but their autosegmentation volumes differed by less than 8% (338uL and 315 mm^3^, not significant).

Theoretically, subtracting a pre-infusion image from the post-infusion image would remove the background structures and also eliminate the need for the operator-defined box. Subtracting images may be particularly beneficial for autosegmenting T2-weighted images, which have been proposed as an alternative that eliminates the need to co-infuse a Gadolinium-based contrast agent [15], [23], [24]. Without subtraction, hyperintensities on T2-weighted MRI images resulting from disease pathology such as pre-existing peritumoral edema could be mistaken for infusate [23].

However, subtracting a pre-infusion image adds a level of complexity that might be clinically impractical in protocols that only acquire a post-infusion image or move the patient between scans, requiring image alignment that could seriously contaminate the image subtraction and correction of edge- or through-plane artifacts. A preferable strategy might be to autosegment and remove the confounding structures or to run a brain-extraction algorithm prior to using the semi-automated tool.

Future studies should adapt the algorithm for FLAIR MR imaging, which has recently been applied to detect Gadolinium-labeled compounds in the CSF [25], [26], as well as to other imaging modalities used to track distributions such as CT [27] and SPECT [4]. These applications may require adjusting the starting values and threshold applied in this study. Studies comparing the output of the segmentation tool to post-mortem histology should validate the edge on the shoulder of the infusion (Figure 3C) and address whether free Gadolinium reasonably approximates the distribution of large drugs [25], [26], despite the discrepancy in molecule size, or whether the distributions are different [28] and necessitate using larger contrast agents like liposomal Gadolinium-DTPA [29] or Gadolinium-bound albumin [25].

This tool is applicable to numerous upcoming CED trials using adeno-associated virus serotype 2 to deliver aromatic l-amino acid decarboxylase (AAV2-AADC) to treat Parkinon’s disease [30], [31], [32], glial-derived neurotrophic factor (AAV2-GDNF) to treat Parkinson’s disease [21], [22], a retrovirus to deliver cytosine deaminase for treating brain tumors [33], [34] and liposomal toxins for treating brain tumors [13], [35], [36], [37].

In conclusion, this study has validated the performance of an efficient algorithm to segment infusions by constructing models of infusion pixels and background pixels. The semi-automated tool produced similar volumes to manual segmentation by experienced operators, but significantly reduced inter-operator variability.
